# The Differential Nature of Weak Ties: Race Differences in Adults’ Daily Interactions with Others

**DOI:** 10.1093/geroni/igaf122.2999

**Published:** 2025-12-31

**Authors:** Katherine Fiori, Amy Rauer, Kira Birditt, Angela Turkelson, Oliver Huxhold

**Affiliations:** Adelphi University, Garden City, New York, United States; University of Tennessee, Knoxville, Tennessee, United States; University of Michigan, Ann Arbor, Michigan, United States; University of Michigan Institute for Social Research, Ann Arbor, Michigan, United States; German Centre of Gerontology, Berlin, Berlin, Germany

## Abstract

Recent research has highlighted the importance of interactions with weak ties for well-being. However, structural, cultural, and individual-level racism may influence the ways in which racially minoritized groups (e.g., Black Americans) interact with others, particularly those individuals outside the network (‘weak ties’). Yet most studies on race differences in social ties take a global approach rather than examining daily encounters. Thus, we used ecological momentary assessment and hierarchical network mapping data to examine whether there were race differences in types of daily social interactions (close/in network vs. weak/outside network interactions) and in their valence (positive, negative, ambivalent). Participants from the Stress and Well-being in Everyday Life (SWEL) study (N = 168; M age = 52.96, range = 33-91; 66.1% female; 47.6% Black) completed surveys every three hours for 4-5 days. Results showed that 36.6% of all interactions occurred with people outside of the network. Although White Americans reported having more interactions than Black Americans, the proportions of interactions with close and weak ties did not differ. Interestingly, we found that the proportion of interactions with weak ties was negatively associated with the proportion of positive interactions and positively associated with the proportion of ambivalent interactions for Black Americans only, controlling for age, gender, education, and total number of interactions. Follow-up analyses consider daily experiences of discrimination as a potential mechanism. Findings point to the importance of acknowledging the role of context in shaping the nature and potential effects of daily social interactions with others.

